# Supplementary material for: Mass Transport of Dye Solutions through Porous Membrane Containing Tannic Acid/Fe3+ Selective Layer
Source: Membranes (Basel). 2022 Dec 1;12(12):1216. doi: 10.3390/membranes12121216 (PMC9781510; doi:10.3390/membranes12121216)
Supplement: Supplementary file 1 [file membranes-12-01216-s001.zip › membranes-2062421-supplementary.pdf]

Supporting Information

Mass Transport of Dye Solutions through Porous Membrane Containing Tannic Acid/Fe^{3+} Selective Layer

Hluf Hailu Kinfu [1], Md. Mushfequr Rahman [1,*], Nicolás Cevallos-Cueva [1] and Volker Abetz [1,2]

[1] Helmholtz-Zentrum Hereon, Institute of Membrane Research, Max-Planck-Straße 1, 21502 Geesthacht, Germany; hluf.kinfu@hereon.de (H.H.K.); nicolas.cevallos-cueva@hereon.de (N.C.-C.); volker.abetz@hereon.de (V.A.)
[2] Institute of Physical Chemistry, University of Hamburg, Martin-Luther-King-Platz 6, 20146 Hamburg, Germany
* Correspondence: mushfequr.rahman@hereon.de; Tel.: +49-4152872446

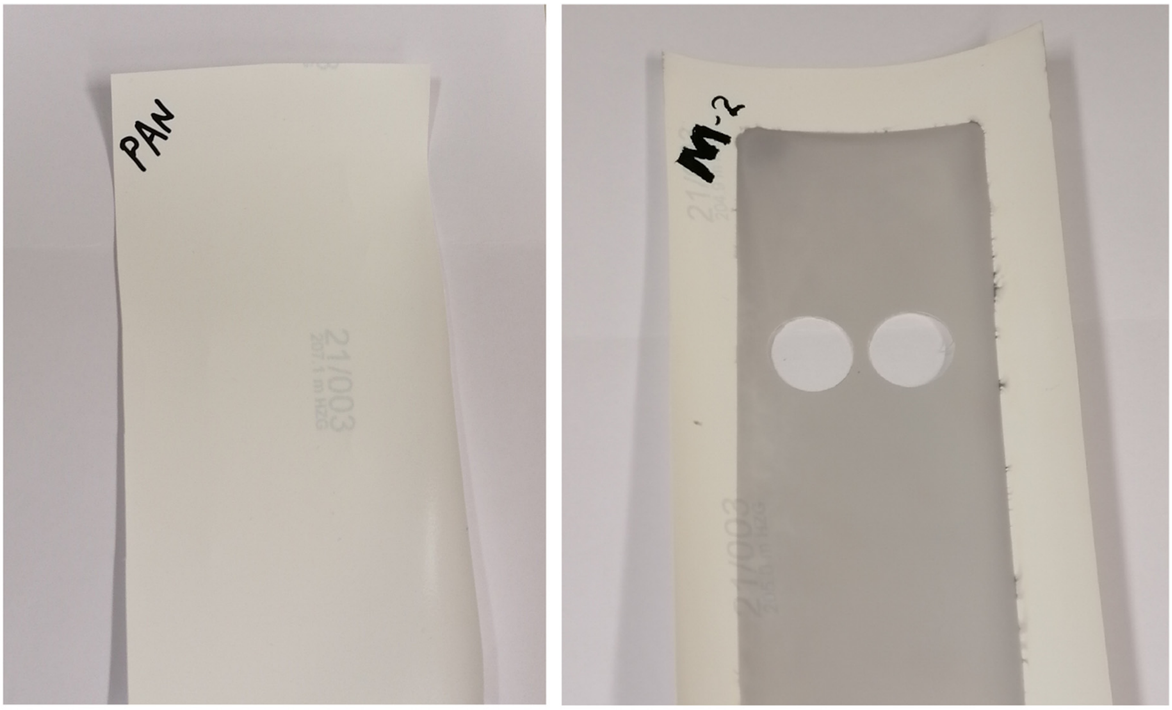

Figure S1. Comparison of photographic images of pristine PAN membrane support and TA-Fe^{3+} membrane used for retention measurement. Color change supports the formation of metal-polyphenol selective layer on top of the porous support as is also confirmed with significant drop in water flux.

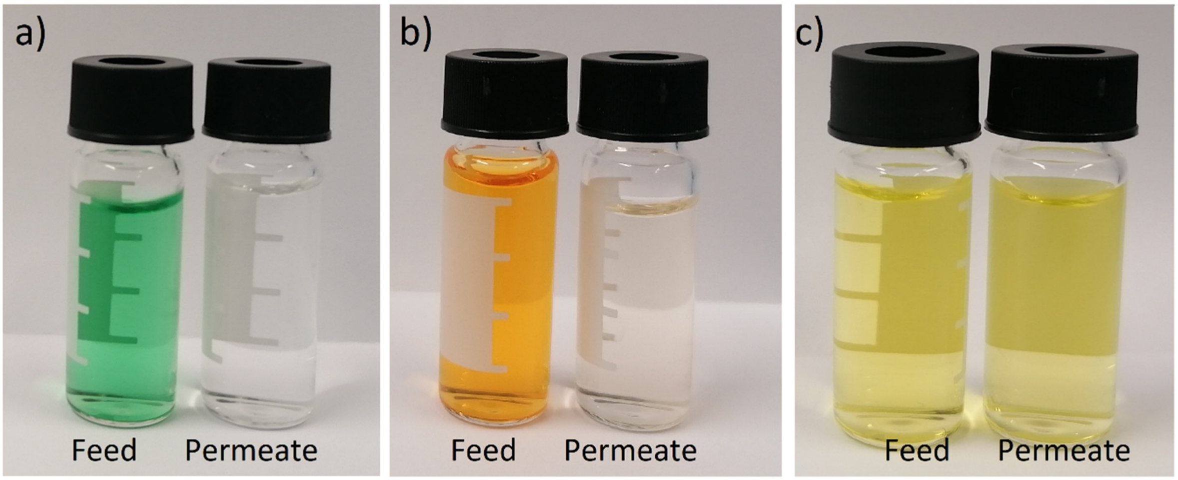

Figure S2. Photographic images of feed and permeate samples from a) naphthol green B, b) orange II and c) riboflavin 0.1mM solution retention tests at 3 bar using M2 membrane

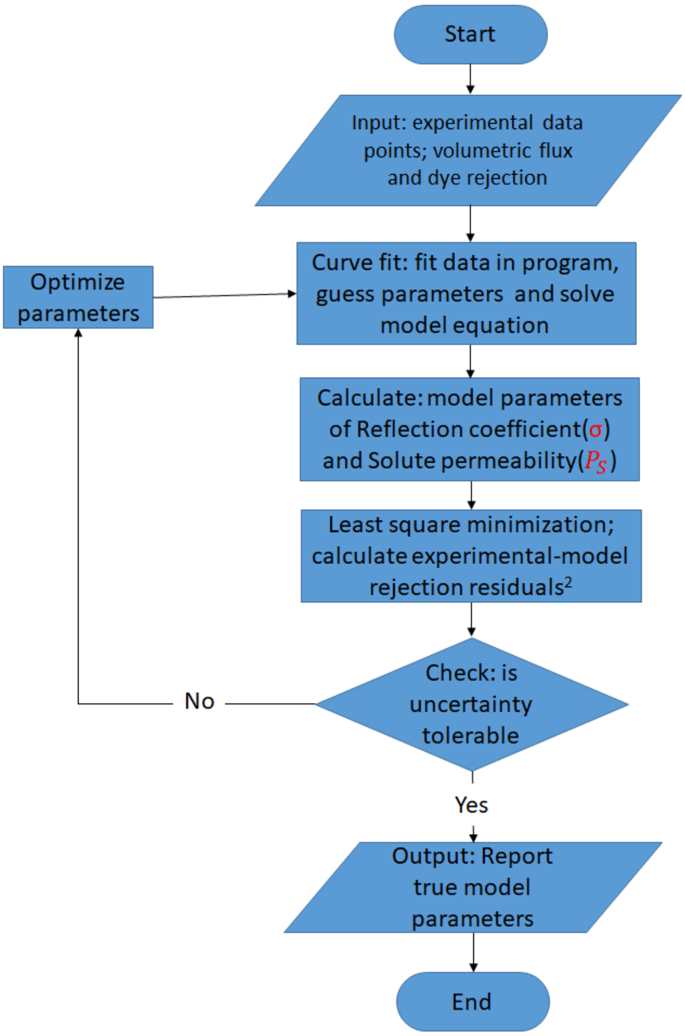

Figure S3. Flow chart of the simulation algorithm loop for solving non-linear equations of Spiegler-Kedem-Katchalsky model in the current study
